# Sensory profile and technological characterization of boneless dry-cured ham with lactulose added as a prebiotic

**DOI:** 10.5713/ajas.19.0152

**Published:** 2019-05-28

**Authors:** Hewerton Barbosa Gomes, Lorena Mendes Rodrigues, Armando Abel Massingue, Ítalo Abreu Lima, Alcinéia de Lemos Souza Ramos, Eduardo Mendes Ramos

**Affiliations:** 1 Food Science Department, Federal University of Lavras (UFLA), Lavras, Minas Gerais, 37200-000, Brazil; 2 Higher School of Rural Development, Eduardo Mondlane University (UEM), Vilankulo, C. P. 1304, Mozambique; 3 Federal Institute of Education, Science and Technology of Bahia (IFBA), Barreiras Campus, Barreiras, Bahia, 47808-006, Brazil

**Keywords:** Check-all-that-apply Analysis, Pork Product, Sensorial Analysis, Texture Profile Analysis, Instrumental Color

## Abstract

**Objective:**

This study investigates the technological and sensory profile of boneless dry-cured ham with different contents of lactulose added as a prebiotic ingredient.

**Methods:**

In addition to the control samples (without the addition of lactulose), three treatments were formulated to contain 2%, 4%, or 6% lactulose. Technological (lactulose content, instrumental color and texture profile analysis) and sensory (acceptance and check-all-that-applies tests) analyses were performed on the final product.

**Results:**

The lactulose content in the finished product (1.86%±0.23%, 3.16%±0.18%, and 2.51%±1.35%) was lower than the lactulose originally added (2%, 4%, and 6%, respectively). The addition of 4% and 6% lactulose made (p<0.05) the products darker (lower L*) and redder (lower *h*) with higher hardness and chewiness values, when compared to control samples. The additions of 2% and 4% lactulose reduce the appearance acceptability of the products, but overall the treatments were well accepted.

**Conclusion:**

The use of up to 4% lactulose as a prebiotic in the production of boneless dry-cured hams provides an alternative to improving its nutritional value with little alteration in the technological characteristics and still meeting the sensory characteristics desired by consumers.

## INTRODUCTION

Currently, in the food sector there has been a great demand for healthier and more functional products, mainly due to the greater concern of consumers with the nutritional quality of what they are ingesting. One product in this category is prebiotics, which are non-digestible food ingredients that, when passing through the upper gastrointestinal tract, are selectively fermented to stimulate bacteria in the gut [1].

The production of functional foods containing prebiotic ingredients is an area that has been featured in the food industry throughout the years and is a very promising market not only for economic reasons, but also because of scientific evidence of its benefits [2]. However, functional meat products still constitute a very little used field in the sector, mainly because they are in their initial phase of development, as they are relatively new in the market. Functional meat products are more complex than non-meat products because of the need for adjustments between the technological, safety and quality aspects and the health benefits of such foods [3]. Moreover, the use of these ingredients in meat products still needs to be studied further to make a greater variety of products available in this segment.

Recently, some researchers suggested the use of lactulose as a functional ingredient in meat products, such as cooked hams [4], dry-cured hams [5] and fermented sausages [6]. Lactulose is a disaccharide consisting of glucose and fructose (4-O-β-D-galactopyranosyl-D-fructose), which is neither metabolized nor absorbed in the small intestine, and thus, it is available for bacteria in the large intestine, where it is metabolized preferentially by bifidobacteria and lactobacilli [7]. In addition, lactulose reduces cholesterol, attenuates the blood glucose level and improves the metabolic absorption of certain minerals, such as calcium, magnesium, and iron [8].

Among the products with the potential to be combined with lactulose, boneless dry-cured hams seem to be a good alternative since in Brazil they are considered noble, for a consumer with higher purchasing power, and pleases the international market. Traditionally, dry-cured hams have been marketed as a whole piece, but the product demands a very long manufacturing time due to the need for an extended period of drying, which can last from 9 to 24 months [9]. To overcome this problem, the ripening process has been accelerated by using deboned meats offering advantages during the production, storage and transport because it decreases the piece weight and volume in a quantity equivalent to the size of the bone [10], being more suitable for cutting and slicing operations. However, the use of deboned meats to make restructured uncooked products requires the application of a cold-set binding agent, such as the enzyme transglutaminase [5,11–14]. The transglutaminase catalyses acyl transfers in reactions between γ-carboxyamide groups of glutamine residues and ɛ-amino groups of lysine residues of proteins, promoting protein aggregation and improved texture in muscle foods [15].

The preparation of boneless dry-cured hams also facilitates the incorporation of functional ingredients. Lima et al [5] have been able to produce a boneless dry-cured ham with 1.44% lactulose, without significant differences in the physicochemical or technological characteristics when compared to the control (without lactulose). Although the lactulose content (0.58 g/portion) is well below those recommended by the Brazilian legislation [16] to be considered a “source of dietary fibre” (2.5 g/serving) or “high dietary fibre content” (5.0 g/portion), the feasibility of using this prebiotic in the manufacturing of boneless dry-cured hams was proven.

In addition to contributing to human health, products with functional claims need to have commercial value, and therefore, the main requirements remain the same as for any other meat product; the sensory and technological aspects of the final product must be satisfactory [6]. Therefore, the aim of this study was to establish the sensory profile and technological characterization of boneless dry-cured hams, based on the process of Lima et al [5], with higher contents of lactulose added as a prebiotic source.

## MATERIALS AND METHODS

### Materials

Frozen pork hams with the Federal Inspection seal were obtained at the local market, brought to the Laboratory of Meat Science and Technology (Lab Carnes) at the Federal University of Lavras (UFLA), thawed (4°C/24 h) and manually deboned immediately before processing. Lactulose syrup (667 mg/mL of lactulose; Farmasa, São Paulo, SP, Brazil) was used as the prebiotic, and D-(+)-gluconic acid δ-lactone (glucono delta-lactone, GDL; Sigma-Aldrich, San Luis, MO, USA) was used as an acidulant. The tranglutaminase Activa GS was donated by Ajinomoto do Brazil (Ajinomoto Co. Inc., Chuo, Tokyo, Japan), and the other additives were donated by New Max (New Max Industrial Ltd.; Americana, SP, Brazil).

### Ham formulation and processing

The following four formulations (treatments) were prepared: without lactulose (CONT) and manufactured with the addition of 3%, 6%, and 9% lactulose syrup in order to contain 2%, 4%, and 6% lactulose (LAC2, LAC4, and LAC6) respectively.

**Figure 1:**
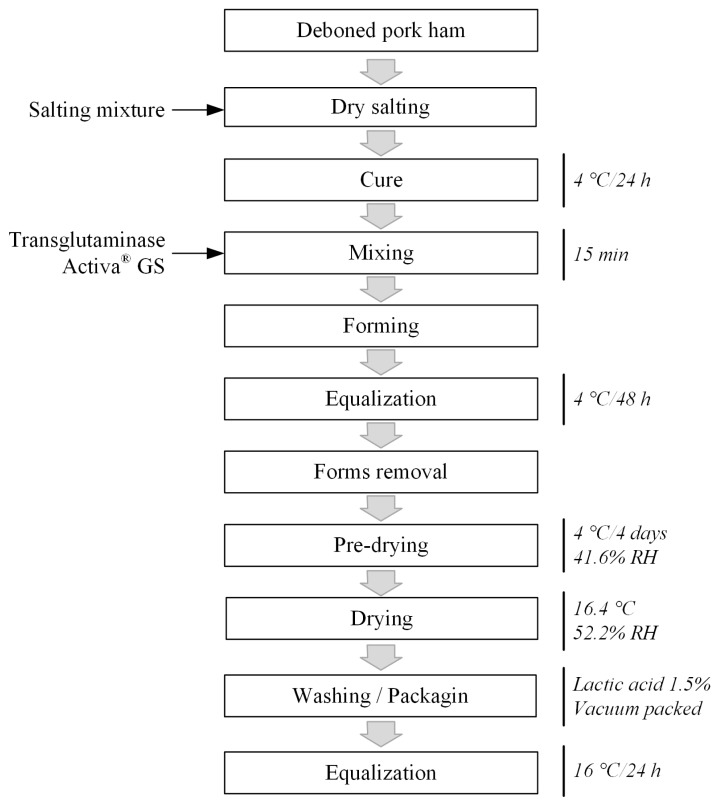
Flowchart of the process for producing boneless dry-cured ham.

Boneless dry-cured hams were manufactured according to Lima et al [5], as illustrated in Figure 1. As the salting mixture, 3% sodium chloride, 150 ppm sodium nitrite, 300 ppm sodium nitrate, 0.25% glucose, 0.25% sucrose, and 0.3% GDL were used. The GDL was used to accelerate the ripening process [13]. The salting mixture (3.845% salts and 3%, 6%, or 9% lactulose syrup in the LACT treatments) was distributed manually over the pork and maintained at 4°C for 24 hours for the initial dry-curing process. After that, the salted/cured pork was mixed (model MJ35; Jamar Industries Ltd., Sao Paulo, SP, Brazil) with a 0.5% solution of tranglutaminase Activa GS 25% for 15 min, formed into 1 kg metallic forms and maintained at 4°C for another 48 hours for finishing the dry-curing process and equalization. The product was removed from the metallic forms and submitted to the pre-drying and drying processes in a climate chamber (model EL202; Electrolab, SP, Brazil) with controlled temperature. The air relative humidity (RH) in the chamber was not controlled but accompanied with a thermohygrometer (model Data Logger HT500; Instrutherm, RJ, Brazil). In the pre-drying (post-salting) process, the products were maintained for 4 days at 4.1°C±0.6°C (41.6%±6.9% RH). Then, the products were covered with a lard paste (with 2% sodium chloride) to avoid excessive drying and maintained at 16.4°C±0.5°C (52.2% ±10.9% RH) for drying and to finalize the ripening process. During the entire process, the products were weighed periodically, and upon reaching 40% weight loss, the pieces were washed with distilled water (±40°C) and 1.5% lactic acid solution, dried with paper towels, packed in a vacuum and finally maintained at 16°C for 24 hours for final equalization. The mass loss during processing was calculated and expressed as a percentage.

### Technological analyses

The water activity (aw) of the dry-cured hams was measured at 25°C using an AQUALAB Lite dielectric hygrometer (Decagon Devices Inc., Pullman, MA, USA).

The lactulose concentration in the finished products was determined by the spectrophotometric method proposed by Zhang et al [17] and adapted by Lima et al [5] for meat products. Briefly, 5 g meat was homogenized (Turratec TE 102; TECNAL, Piracicaba, SP, Brazil) in 50 mL of distilled water, filtered on quantitative Whatman No. 1 filter paper and diluted to 100 mL. After 1:50 dilution, a 1.0 mL aliquot was mixed with 2.8 mL of sulfuric acid (75%) and kept in a water-bath at 46°C for 5 min. Then, 0.2 mL of cysteine-tryptophan hydrochloride solution (25 mg/mL cysteine and 0.8 mg/mL tryptophan in 0.01 M HCl) was added, mixed in a vortex mixer and maintained at 46°C for an additional 70 min. The tubes were cooled in running water for 5 min, and the absorbance was read at 518 nm in a digital spectrophotometer (Kasuaki IL-227; São Paulo, SP, Brazil). The lactulose concentration was determined from an analytical curve elaborated with standard lactulose solution (5 to 25 μg/mL; Sigma-Aldrich, USA) and expressed as a percentage.

Meat color was assessed using a Minolta CM-700 (Konica Minolta, Japan) colorimeter as described by Lima et al [5], using the CIELAB system in the specular component excluded mode with a D65 standard illuminant, an observer angle of 10° and an 8 mm aperture. Six measurements representing the entire internal cross-sectional surface were taken from each sample. The lightness (L*), redness (a*), and yellowness (b*) were recorded. The angular coordinates of chroma (C*) and hue angle (*h*, degrees) were calculated using the following formulas [18]: C* = (a*2+b*2)1/2 and *h* = arctan (b*/a*).

The texture profile analysis was carried out using a universal Texture Analyser TA.XT2i (Stable Micro Systems Ltd., Godalming, UK), as described by Ramos and Gomide [18]. Six cubes (with 10 mm edge cores) were obtained from each sample and compressed twice to 50% of their original height at room temperature with a compression flat cylindrical aluminium probe (36 mm diameter). A crosshead speed of 180 mm/min was applied. There was no rest time between the two cycles of compression. Force time curves were recorded during compression, and the following five texture attributes were calculated: i) hardness (N), peak force required for the first compression; ii) springiness (mm), distance sample recovers after first compression; iii) adhesiveness (N mm), the negative force area for the first bite representing the work necessary to pull the compressing plunger away from the sample; iv) cohesiveness, ratio of positive force area during the second compression to that in the first compression; and v) chewiness (N mm), the product of the hardness, cohesiveness and springiness.

### Sensory evaluation

Sensory evaluation was carried out after approval of the Research Ethics Committee of UFLA and under protoregistration (protocol CAAE 30774814.3.0000.5148) at the National Research Ethics System (SISNEP, São Paulo, SP, Brazil).

To describe the sensory characterization of each formulated product, the check-all-that-apply (CATA) questions were evaluated according to Ares et al [19], with adaptations for meat products described by Jorge et al [20]. The test was performed in two stages. First, the CATA questions were defined by 10 randomly recruited untrained participants, consisting of undergraduate and graduate students from UFLA. Slices with a thickness of 3 mm (~20 grams) of each product were presented in a single test session (network technique), in which the judges used open questions to establish the appropriate terms to describe the color, appearance, flavor, odor, and texture of the products. The most mentioned terms for each attribute (Table 1) were chosen to compose the CATA questions.

In the second stage, 60 untrained participants (68% women and 32% men), consisting of professors as well as undergraduate and graduate students, were randomly recruited from the UFLA. This group were predominantly young individuals (91% between 18 and 30 years old and only 9% between 31 and 45 years old). The sensory analysis was performed during a single testing session conducted in individual cabins with white light. The 3-mm thick sliced samples were labelled with a 3-digit code and were presented to each panelists randomly and balanced in a monadic sequence. Mineral water was offered for cleaning the palate between the samples. The panelists received the sensory evaluation form (acceptance test) and evaluated the samples using a 9-point hedonic scale (“1, disliked it very much” to “9, liked it very much”) for each attribute (appearance, flavor, odor, texture, and overall impression). In the same form, the panelists were asked to check all the CATA terms (Table 1) that they considered to appropriately describe each attribute.

### Statistical analyses

To characterize the products, the technological analysis data were tested using the F-test (analysis of variance [ANOVA]), in a completely randomized design with four treatments (samples) and three replicates; when significant (p<0.05), the means were separated using the Tukey test.

For the acceptance test, a statistical analysis was performed by assuming a randomized block design, in which each assessor represented a block. The data were tested by the F-test (ANOVA), and when the result was significant (p<0.05), the means were separated using the Tukey test. In addition, each attribute was analysed individually with an internal preference map (IPM), and the attributes of appearance, flavor, odor, and texture were simultaneously analysed by a three-way internal preference map (IPM tri-plot), which is also known as parallel factor analysis (PARAFAC) according to Nunes et al [21].

To identify the relationships between the CATA terms selected for each sample, an external preference map (EPM) was used [20]. The EPM analysis was based on the regression of external descriptors against the overall impression of each consumer’s data. To generate the EPM map, a significance of 30% was considered, as proposed by Elmore et al [22].

Statistical analyses were performed using the SAS statistical package, version 9.2 (SAS Institute Inc., Cary, NC, USA). The IPM and EPM analyses were performed using the SensoMaker statistical software package version 1.91 (Lavras, Brazil).

## RESULTS AND DISCUSSION

After a pre-drying process (4 days of processing at 4°C), the average mass loss was 15.07%±1.44%, but at the end of 36 days of ripening (16°C), an average total loss of 40.02%±2.23% was achieved. The average value of the water activity (aw) of finished products was 0.91±0.01, slightly above that (0.90) reported by Lima et al [5] in boneless dry-cured hams made with 2% lactulose. The aw observed in this experiment was slightly below the maximum (0.92) required by Brazilian legislation for dry-cured hams [23] and exactly the 0.91 reported by Fernández-Salguero [24] to be considered a stable product without the need refrigerated storage.

### Technological characteristics

The effects of lactulose addition on the technological characteristics of boneless dry-cured hams are described in Table 2.

The control treatment presented residual values of this disaccharide, probably due to interference from the aldoses present [17], such as the added glucose, or even an intrinsic analytical variation of the method itself, especially in the extraction step [6]. Other authors have also observed residual values of lactulose in dry-cured ham (0.17%) [5] and fermented sausage (0.37%) [6] samples that were not from the addition of this prebiotic.

As expected, the lactulose content was higher in samples with greater quantities of this prebiotic added, although there was no difference (p>0.05) between dry-cured hams added with 4% and 6% lactulose. This may be justified by the observation of a large amount of liquid lost during the formation step in the preparation of treatments containing 6% lactulose. In this treatment, a large amount of syrup (90 mL/kg) was necessary to achieve the amount of lactulose required, and it was most likely expelled with the pressure exerted on the product during forming into metallic forms.

Another observation is that the average values of lactulose content in the finished products were lower than the amount of lactulose originally added. Moreover, considering that during the drying process the products lost approximately 40% of their mass, mainly by the evaporation of water, an increase in the solute concentration was expected and consequently an increase in the lactulose content. This difference was also observed by Lima et al [5] in dry-cured hams (1.44%) and by Coelho et al [6] in fermented sausage (1.27%) with 2% lactulose added. These authors suggested that these differences indicate a possible use of lactulose as a source of carbon by lactic acid bacteria, since lactulose has been reported as one of the most effective substrates for the growth of all eight strains of *Lactobacillus* [25,26].

Even in the treatments containing higher amounts of lactulose (4% added), the amount of lactulose delivered per serving (1.26 g/40 g) was still below the minimum required (2.5 g/40 g) by the Brazilian legislation [16,27] for the product to be considered a “source of dietary fibre”. However, Oliveira et al [4] emphasized that the application of lactulose as a prebiotic in food is restricted to low doses since in high amounts, it acts as a laxative (transitory laxative threshold was 0.26 g/kg body weight). In addition, studies suggest that the consumption of lactulose to exert prebiotic action in adults is effective at much lower concentrations (4 to 10 g/d) than the transitory laxative threshold [28]. According to Terada et al [29], 3 g of lactulose for two weeks was able to significantly increase the number of bifidobacteria in the faecal flora. This amount could almost be achieved with two servings of dry-cured ham formulated with 4% lactulose. Moreover, as pointed out by Coelho et al [6], considering the low daily consumption of these products, there is no expectation of these being used as a single source of fibre in the diet but rather that they may contribute to the daily consumption of this type of ingredient.

With regards to the chroma (C*) values, the addition of 2% lactulose did not change (p>0.05) the lightness (L*) and hue (*h*) color of samples, which was also observed by Lima et al [5] for the same product. However, in this experiment, the dry-cured hams added with 2% lactulose were darker (lower L*) and reddish (lower *h*), although with more intense color (higher C*), than the products (L* = 45.30, C* = 7.00, and *h* = 45.17) made by those authors. Overall, the addition of amounts of lactulose greater than 2% increased these differences, reducing the L* and *h* values of the products.

For the texture profile attributes, the same behaviour was observed, with samples with 4% or 6% of lactulose added having higher (p<0.05) hardness and chewiness values than the control ones. Otherwise, the cohesiveness, adhesiveness and springiness did not change (p>0.05) with treatments. These changes were also observed by Oliveira et al [4] when evaluating hams with higher additions of lactulose (3%, 6%, and 10%). According to these authors, higher hardness and chewiness with the addition of lactulose could be due to the saccharides (glucose and galactose) and disaccharides (lactose) present in the syrup, which could retain water molecules forming colloidal solutions, which changes the texture of a system.

### Sensory profile

For sensory profile of the dry-cured hams, the addition of lactulose as a prebiotic only affected (p<0.05) the appearance of the products (Table 3). The addition of 2% of lactulose reduced the appearance acceptance of the products compared to the control sample (without lactulose), but the appearance scores of the products containing lactulose increase with increasing amounts of prebiotic added. In fact, samples with higher additions (6%) of lactulose did not differ (p>0.05) from the control samples, these being more preferred by the panelists.

In general, all sensory acceptance attributes were well-accepted by the panelists, which reported the perceptions between “I liked it moderately” and “I liked it very much”. With the exception for the appearance, these results agree with Coelho et al [6], who reported no differences in the acceptance scores of dry-cured sausages elaborated without and with 2% lactulose.

**Figure 2:**
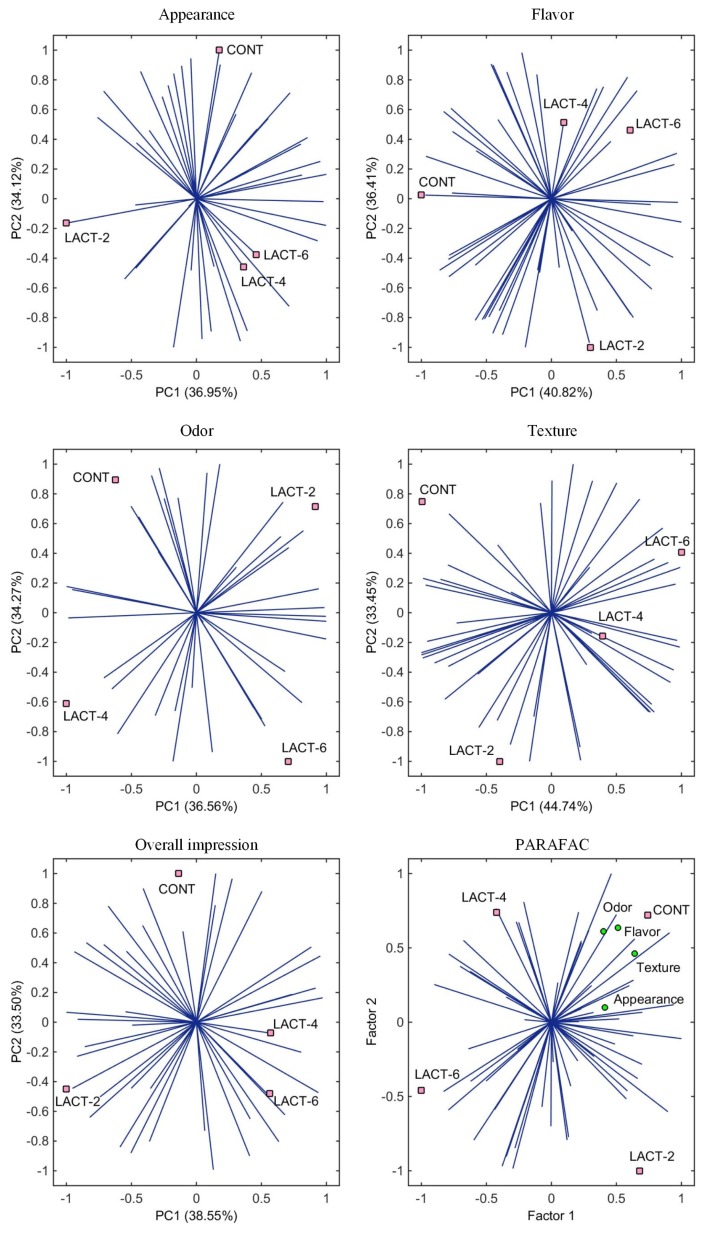
Internal preference maps (IPM) and PARAFAC graph for the sensory attributes (appearance, flavor, odor, texture, and overall impression) based on their consumer scores (shown as vectors in the plot) of the elaborated boneless dry-cured hams. CONT, samples without lactulose; and LACT-2, LACT-4, and LACT-6, samples with 2%, 4%, and 6% of lactulose added, respectively.

The acceptance of the products was also evaluated by an IPM of each product attribute (appearance, flavor, odor, texture, and overall impression). For all attributes, the first two principal components (PCs) of the IPM explained more than 70% of the variance in the data (Figure 2). In the IPM graphs, the vectors represent the consumers’ liking scores for the products in a two-dimensional space; high density of vectors (panelists scores) in the direction of a sample indicate a greater preference. Accordingly, it is possible to observe in the IPM graphs the dispersion of the scores for the attributes flavor, odor, texture, and overall impression, which indicates an absence of preference by the consumers. For the appearance of the products, it is clearly perceived that samples containing 2% lactulose (LACT-2) were the least preferred and samples without lactulose (CONT) were the most preferred.

The consumer acceptance data were also organized into a three-way IPM array, named PARAFAC, where the correlations between the samples, consumers (vectors) and sensory attributes are shown simultaneously on a single graph (Figure 2). The core consistency diagnostic (CORCONDIA) of the PARAFAC model explained 70.34% of the correlation between the two PCs. By the PARAFAC graph, all evaluated attributes were positively highlighted (more preferred) in the control sample (CONT) than in samples with lactulose added. However, according to Jorge et al [20], the graphical representation of PARAFAC provides less information for each attribute than the individual analysis of the IPM, especially the IPM for the overall impression, since each consumer weighted the sensory attributes differently when determining his or her overall impression.

**Figure 3:**
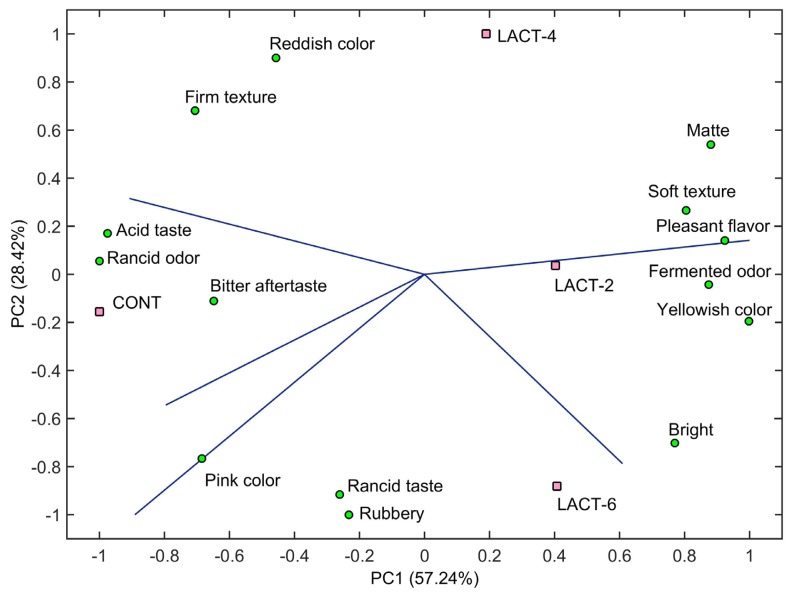
External preference map (EPM) of the sensory terms on the check-all-that-apply (CATA) questionnaire for the elaborated boneless dry-cured hams in the correlation matrix with the overall consumer impression. CONT, samples without lactulose; and LACT-2, LACT-4, and LACT-6, samples with 2%, 4%, and 6% lactulose added, respectively.

A better understanding of how these sensory attributes were affected by the treatments can be obtained through the EPM of the CATA analysis, associating the consumer preference with the product characteristics (descriptive attributes). The EPM was generated from the number of times that the consumers associated each of the 14 sensory terms of the CATA questions (Table 1) with the samples and overall impression scores from acceptance test. The PC plots show the relative positions of the samples and factor loadings indicate the attributes that best describe the dimensions of the perceptive space. Only the slopes for 6 panelists (of the 60 untrained participants) that provided valid models (p≤0.30; [22]) were plotted on the map (Figure 3). Despite the low number of valid responses (consumers), the two main components (PC1 and PC2) of the EPM explained 85.66% of the variance in the data after fitting with a vector model with a coefficient of determination (R^2^) of 0.98. According to Ares et al [30], CATA is useful for identifying consumer perceptions, even when there are not large differences between the samples, being a valuable tool for understanding consumers’ perception of sensorial and hedonic characteristics.

Like the PARAFAC from the acceptance test, the EPM enabled the spatial separation of samples in four distinct groups, each with specific attributes. However, by the PC1 (57.24% of the variance), two groups could be distinguished: samples with lactulose (LACT-2, LACT-4, and LACT-6); and samples without lactulose (CONT). Overall, samples with lactulose added were grouped mainly by the perception of a soft texture, fermented odor, pleasant flavour, and yellowish color. In these samples, a slightly sweet taste could be expected due to the presence of lactulose, whose sweetness was estimated to be 48% to 62% of that of sucrose [31]. Despite this, during the first panel section, wherein 10 panelists used an open-ended question to establish the appropriate terms for describing their characteristics for each sensorial attribute (CATA terms), no panelists noticed the perception of sweet taste in the products. Even so, the term “pleasant flavor” described by the panelists may be related to the possible sweetness in the samples with lactulose. Moreover, the absence of sweetness perception may be associated with a higher acidity described in the control samples. Something similar was also observed by Coelho et al [6], in which consumers related the term “sweet taste” to samples of fermented sausages formulated with and without 2% lactulose as prebiotic, but this relation was not verified when these samples were added from the probiotic *L. paracasei*; the fermented sausages with the probiotic were positively highlighted with the term “acid taste”. In restructured cooked hams, Oliveira et al [4] reported that the sweet taste was perceived in the samples added of 6% and 10% of lactulose, but not samples with 3% lactulose.

Finally, the differences observed in the appearance attributes (Table 3; Figure 2), with lower scores for samples with 2% lactulose added, seem to be related to the perception of a yellowish coloring in this sample. By PC2, dry-cured hams with 4% lactulose added were perceived with more matte and redder color and those with 6% lactulose added were perceived as brighter than samples with 2% lactulose.

## CONCLUSION

It was possible to elaborate boneless dry-cured hams with higher amounts of lactulose added as a prebiotic, while maintaining the technological and sensory profile such as those of the traditional products. The addition of up to 4% lactulose provided products with higher concentrations of this prebiotic, improving its nutrition value, with little alteration in the technological characteristics and meting the sensory characteristics desired by consumers.

## Figures and Tables

**Table 1:** Terms surveyed for check-all-that-apply (CATA) questions of boneless dry-cured hams elaborated with and without lactulose according to each sensory attribute

Appearance	Odor	Flavor	Texture
Bright	Rancid odor	Rancid taste	Firm
Matte	Fermented	Acid taste	Soft
Yellowish color	-	Bitter aftertaste	Rubbery
Pink color	-	Pleasant	-
Reddish color	-	-	-

**Table 2:** Mean values (±standard deviation) of the technological characteristics of boneless dry-cured hams elaborated with (LACT) and without (CONT) lactulose

Characteristic	CONT	LACT			Mean

		2%	4%	6%	
Lactulose (%)	0.11±0.05^a^	1.86±0.23^b^	3.16±0.18^c^	2.51±1.35^c^	1.91±1.31
CIE color					
Lightness (L*)	38.64±3.36^a^	38.44±4.57^a^	32.52±2.11^b^	32.72±2.56^b^	35.58±3.42
Chroma (C*)	13.43±1.74^a^	10.44±1.61^b^	10.71±1.12^b^	11.26±1.16^b^	11.68±1.20
Hue (*h*, graus)	28.69±3.64^a^	28.36±7.19^a^	23.95±4.53^b^	24.12±4.67^b^	27.31±4.09
Texture profile					
Hardness (N)	3.51±0.15^a^	3.87±0.60^ab^	4.09±0.87^b^	4.05±0.74^b^	3.88±0.27
Cohesiveness	0.78±0.21	0.82±0.11	0.82±0.11	0.79±0.12	0.80±0.22
Adhesiveness (N×mm)	0.21±0.08	0.30±0.15	0.25±0.12	0.33±0.20	0.27±0.28
Springiness (mm)	5.57±0.60	5.28±0.20	5.98±0.33	5.24±0.47	5.52±0.43
Chewiness (N×mm)	15.25±1.63^a^	16.67±2.11^ab^	20.06±4.51^b^	19.87±3.78^b^	17.97±2.39

CONT, samples without lactulose; LACT, samples with 2%, 4%, and 6% of lactulose added, respectively.

a–c Means with different letters in a row differ (p<0.05).

**Table 3:** Scores1) (mean±standard deviation) from consumer sensory panel assessments for boneless dry-cured hams elaborated with (LACT) and without (CONT) lactulose

Attributes	CONT	LACT			Mean

		2%	4%	6%	
Appearance	7.22±1.28^a^	6.67±1.34^c^	6.83±1.40^bc^	7.10±1.17^ab^	6.95±1.30
Flavor	6.33±1.80	6.37±1.95	6.12±1.78	6.03±1.96	6.21±1.87
Odor	5.88±1.95	6.15±1.96	5.83±1.81	5.87±1.84	5.93±1.89
Texture	6.23±1.75	6.82±1.60	6.53±1.59	6.28±1.79	6.47±1.68
Overall Impression	6.47±1.53	6.53±1.53	6.27±1.69	6.40±1.55	6.42±1.58

CONT, samples without lactulose; LACT-2, LACT-4, and LACT-6, samples with 2%, 4%, and 6% of lactulose added, respectively.

1) From a 9-point hedonic scale: 1 = “disliked extremely”; 5 = “neither liked/disliked”; and 9 = “liked extremely”.

a–c Means with different letters in a row differ (p<0.05).
